# Comparative Study of the Susceptibility to Oxidative Stress between Two Types of Mycobacterium bovis BCG Tokyo 172

**DOI:** 10.1128/mSphere.00111-21

**Published:** 2021-03-10

**Authors:** Keiichi Taniguchi, Daisuke Hayashi, Naomi Yasuda, Mao Nakayama, Kaori Yazawa, Shouta Ogawa, Yuji Miyatake, Saki Suda, Haruka Tomita, Miki Tokuda, Saotomo Itoh, Jun-ichi Maeyama, Naoya Ohara, Saburo Yamamoto, Shigeaki Hida, Kikuo Onozaki, Takemasa Takii

**Affiliations:** a Department of Molecular Health Sciences, Graduated School of Pharmaceutical Sciences, Nagoya City University, Nagoya, Aichi, Japan; b Japan BCG Laboratory, Kiyose, Tokyo, Japan; c Department of Mycobacterium Reference and Research, Research Institute of Tuberculosis, Japan Anti-Tuberculosis Association, Kiyose, Tokyo, Japan; d Department of Safety Research on Blood and Biological Products, National Institute of Infectious Diseases, Musashimurayama, Tokyo, Japan; e Department of Oral Microbiology, Graduated School of Medicine, Dentistry and Pharmaceutical Sciences, Okayama University, Okayama, Japan; Washington University School of Medicine in St. Louis

**Keywords:** *Mycobacterium tuberculosis*, BCG vaccine, redox, NADPH oxidase, cytokine

## Abstract

Genomic analysis revealed that the vaccine seed lot of Mycobacterium bovis bacillus Calmette-Guérin (BCG) Tokyo 172 contains two subclones (types I and II), but their phenotypic differences have not been elucidated. In this study, we compared the susceptibility of bacilli types I and II to oxidative stress *in vitro* and within host cells. Notably, the subclones displayed similar superoxide dismutase activity; however, foam height in the catalase test and lysate catalase/peroxidase activity were higher for type I bacilli than for type II bacilli. Additionally, type I bacilli were less susceptible to hydrogen peroxide (H_2_O_2_) than type II bacilli. After exposure to H_2_O_2_, antioxidative stress response genes *katG*, *ahpC*, *sodA*, and *trxA* were more strongly induced in type I bacilli than in type II bacilli. Further, we investigated cell survival in macrophages. Fewer type II bacilli were recovered than type I bacilli. However, in the presence of apocynin, a specific inhibitor of NADPH oxidase, type II recovery was greater than that of type I. The production of interleukin 1β (IL-1β), IL-12 p40, and tumor necrosis factor alpha (TNF-α) was higher in type I bacillus-infected macrophages than in type II bacillus-infected macrophages. The proportions of type I and type II bacilli in vaccine lots over 3 years (100 lots) were 97.6% ± 1.5% and 2.4% ± 1.5%, respectively. The study results illustrated that type I bacilli are more resistant to oxidative stress than type II bacilli. Overall, these findings provide important information in terms of the quality control and safety of BCG Tokyo 172 vaccine.

**IMPORTANCE** This study revealed the difference of *in vivo* and *in vitro* antioxidative stress properties of BCG Tokyo 172 types I and II as one of the bacteriological characteristics. In particular, the bacilli exhibited differences in catalase/peroxidase activity, which could explain their different protective effects against infection. The differences correlated with survival in the host cell and the production of proinflammatory cytokines to protect against infection by Mycobacterium tuberculosis. The proportion of bacilli types I and II in all commercial lots of BCG Tokyo 172 over 3 years (100 lots) was constant. The findings also highlighted the importance of analyzing their content for quality control during vaccine production.

## INTRODUCTION

Globally, more than 10 billion individuals have been inoculated with Mycobacterium bovis bacillus Calmette-Guérin (BCG) since the vaccine was introduced in 1921. The Word Health Organization recognizes four main substrains of BCG as international reference strains: Tokyo 172-1, Russia-I, Connaught 1331, and Moreau (1). We previously reported differences in the immunological and biochemical characteristics of 15 BCG substrains (2, 3), and BCG-Russia, BCG-Moreau, BCG-Birkhaug, BCG-Danish, and BCG-Japan (Tokyo 172) were found to be more tolerant to hydrogen peroxide (H_2_O_2_) exposure than the other substrains (2, 3), suggesting that BCG Tokyo 172 can tolerate oxidative stress.

Prior studies have demonstrated that Mycobacterium tuberculosis is susceptible to H_2_O_2_-induced damage *in vitro* (4–7). During bacterial infection, host cells such as macrophages and neutrophils produce large quantities of reactive oxygen species (ROS) (8). M. tuberculosis produces one catalase enzyme encoded by *katG*, and its product subsequently exhibits catalase, peroxidase, and peroxynitritase activities (9). Additionally, M. tuberculosis contains two superoxide dismutase (SOD) isoforms: (i) SodA (10), containing iron in its active site, and (ii) SodC (11), containing Cu and Zn. Although SodA is constitutively expressed, its expression is elevated upon H_2_O_2_ exposure (12). An antisense gene silencing approach has been effectively used to illustrate that SodA protects M. tuberculosis against superoxide *in vitro* (13).

Calmette previously described smooth (S) and rough (R) colony morphologies for the BCG vaccine (14). Honda et al. reported two bacterial subpopulations in BCG Tokyo 172 (15). They found that two colony types (type I and type II) with different morphologies (S and R) are formed by BCG Tokyo 172. Using multiplex PCR, they demonstrated differences between their genotypes in terms of the region of differences (RDs) and SenX3-RegX3 (15). Further, they recorded different growth rates in culture for the colony types. S colonies exhibit a 22-bp deletion in Rv3405c of the RD16 region (type I), and R colonies did not have this deletion (type II) similar to many other BCG substrains (15). Several studies also reported that culture conditions affect the growth and immunogenicity of BCG vaccine substrains (16–18). In this study, we compared the tolerance of BCG Tokyo 172 types I and II to oxidative stress *in vitro*, their survivability in host cells, and their cytokine profiles in macrophages.

## RESULTS

### Oxidative stress tolerance *in vitro* in BCG Tokyo 172 types I and II.

**FIG 1:**
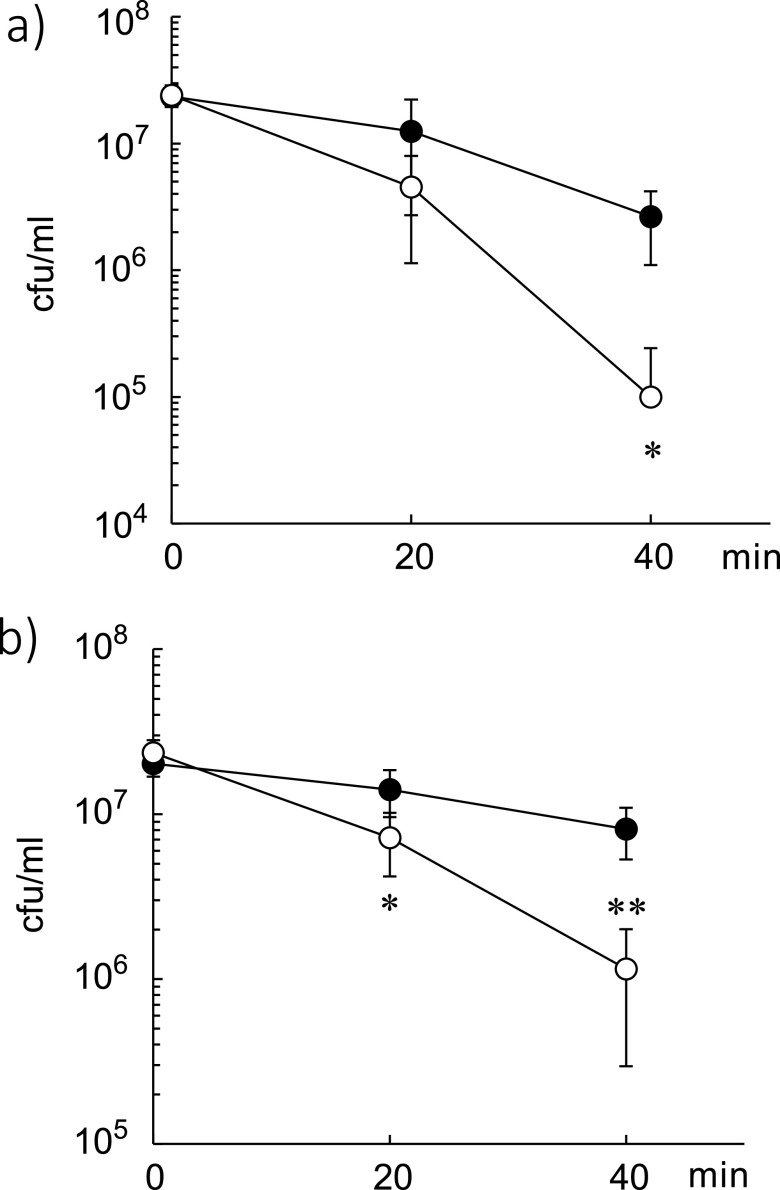
Susceptibility of Mycobacterium bovis bacillus Calmette-Guérin (BCG) Tokyo 172 types I and II to hydrogen peroxide (H_2_O_2_). BCG bacilli were exposed to 20 mM H_2_O_2_ for 20 or 40 min in (a) Sauton medium or (b) Middlebrook 7H9 broth supplemented with albumin and dextrose, and then 10-fold serial dilutions of each medium were made. The diluted bacilli were plated onto Middlebrook 7H11 agar. After 2 or 3 weeks, the bacterial colonies were counted. Solid circle, BCG Tokyo 172 type I; open circle, BCG Tokyo 172 type II. The data are representative of three independent experiments and data are presented as mean and standard deviation. Statistical significance between types I and II was analyzed via one-way analysis of variance, *, *P* < 0.05, **, *P < *0.01.

By investigating the susceptibility of the two types of BCG Tokyo 172 cultured in Sauton medium (Fig. 1a) or Middlebrook 7H9 broth supplemented with albumin and dextrose (AD) (Fig. 1b) to lethal concentration of H_2_O_2_ (20 mM) *in vitro*, we found that the viability of BCG bacilli type I was significantly higher than that of type II after both 20 and 40 min of H_2_O_2_ exposure. The half-lives of bacilli types I and II were 33.2 and 18.1 min, respectively, in Middlebrook 7H9AD broth and 22.2 and 16.4 min, respectively, in Sauton medium. These data suggest that type II bacilli are more sensitive to oxidative stress than type I bacilli.

**FIG 2:**
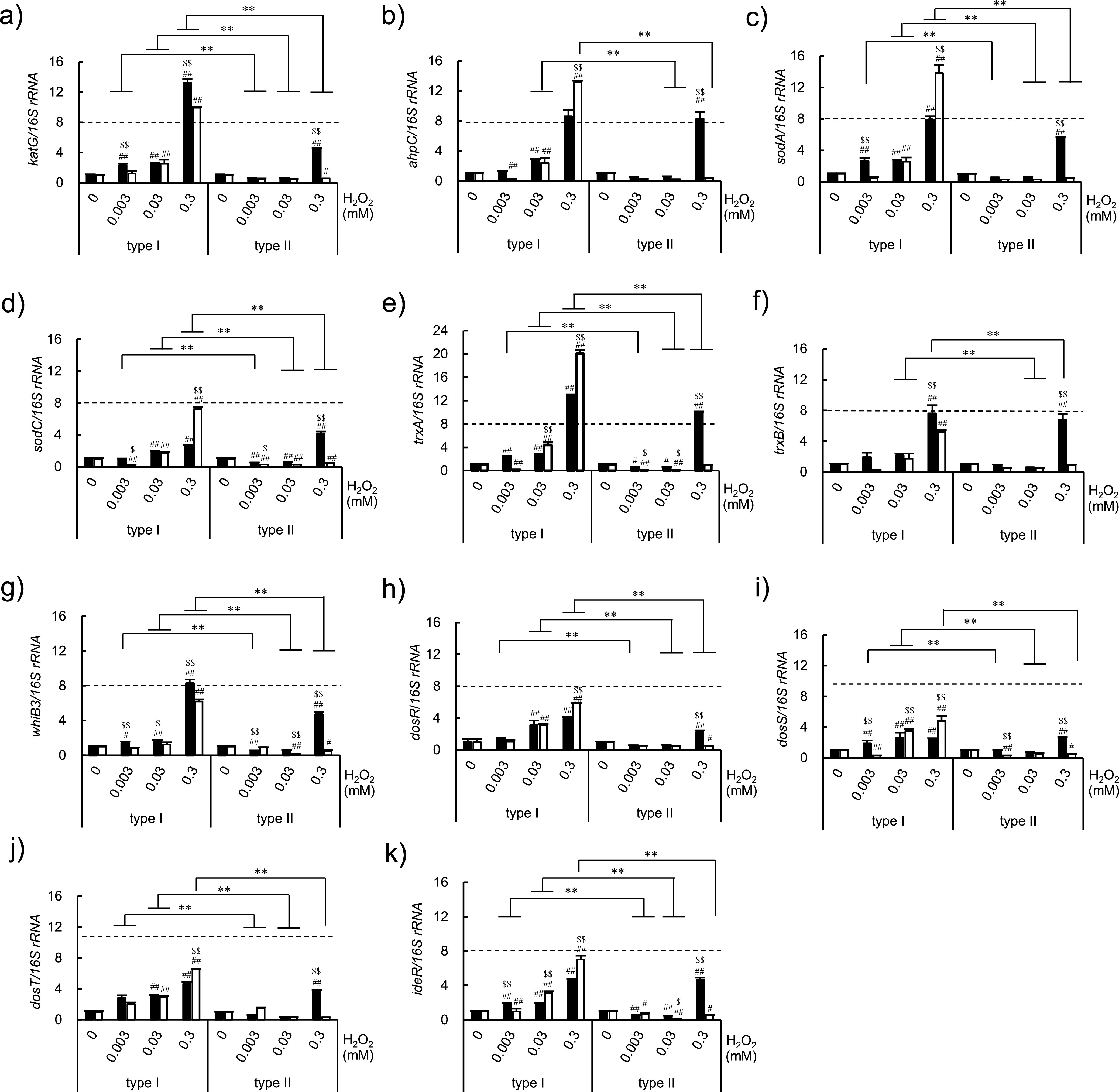
The antioxidative response gene expression in Mycobacterium bovis bacillus Calmette-Guérin (BCG) Tokyo 172 types I and II after hydrogen peroxide (H_2_O_2_) exposure. BCG bacilli cultured with Sauton medium were exposed to H_2_O_2_ (0.003, 0.03, or 0.3 mM) for 5 or 30 min. The mRNA expression of antioxidative response genes was measured by real-time PCR and normalized by that of 16S rRNA without H_2_O_2_ as follows: (a) *katG*, (b) *ahpC*, (c) *sodA*, (d) *sodC*, (e) *trxA*, (f) *trxB*, (g) *whiB3*, (h) *dosR*, (i) *dosS*, (j) *dosT*, and (k) *ideR*. Solid and open columns indicate the result after 5 min and 30 min of exposure to H_2_O_2_, respectively. The data are representative of three independent experiments and the results are presented as mean and standard deviation. Statistical significance among three factors, namely, the type of subclone, exposure time, and H_2_O_2_ concentration, was analyzed by three-way analysis of variance. Statistical significance between types I and II was analysis of variance: type I versus type II, *, *P < *0.05, **, *P < *0.01; versus no H_2_O_2_, ^#^, *P < *0.05, ^##^, *P < *0.01; 5 min versus 30 min, ^$^*P < *0.05, ^$$^*P < *0.01.

Next, we investigated the induction of antioxidative stress response genes by H_2_O_2_ in bacilli types I and II (Fig. 2). The induction of *katG* and *ahpC*, which encode mycobacterial catalase/peroxidase, was significantly stronger in type I bacilli than in type II bacilli (Fig. 2a and b). The mRNA expression of *sodA* and *trxA* was also significantly induced in type I bacilli (Fig. 2c and e). In a previous study, BCG-Japan (Tokyo 172), which contains bacilli types I and II, exhibited the highest catalase activity among various substrains of BCG, and it was most resistant to H_2_O_2_ (3). In the present study, the foam heights produced by a commercial lot of BCG Tokyo 172, BCG Tokyo 172 type I, and BCG Tokyo 172 type II were 15.4 ± 3.5, 12.5 ± 3.6, and 5.8 ± 2.0 mm, respectively (N = 7), indicating a 2-fold difference between types I and II (*P = *0.003, type I versus type II). Thus, increased catalase activity possibly explains the predominance of type I BCG in vaccine lots.

**FIG 3:**
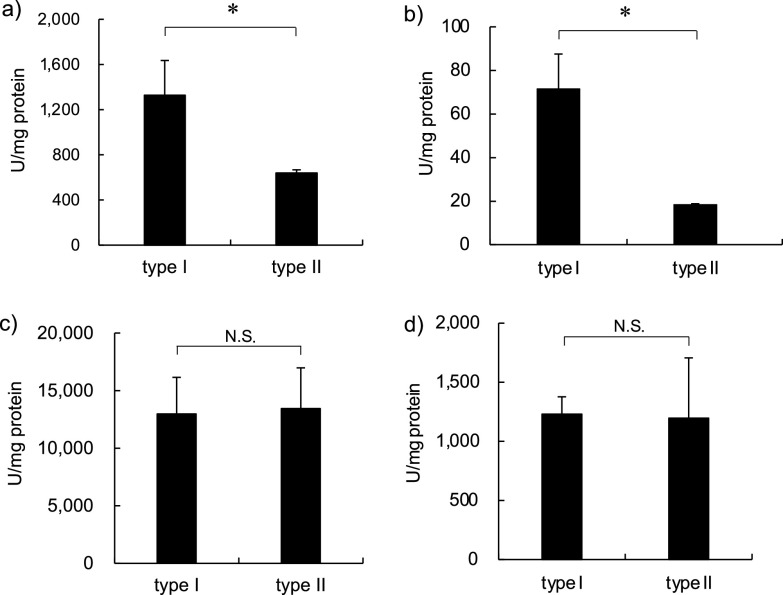
Comparison of catalase and superoxide dismutase (SOD) activities between Mycobacterium bovis bacillus Calmette-Guérin (BCG) Tokyo 172 types I and II. (a and b) Catalase and peroxidase activities in the cell lysates were measured according to the disruption of H_2_O_2_ using a spectrophotometer. (c and d) SOD activity in cell lysates was measured using WST SOD assay kit. The bacilli were cultured in Sauton medium (a and c) or Middlebrook 7H9 broth supplemented with albumin and dextrose (b and d). The data represent three independent experiments and data are presented as mean and standard deviation. Statistical significance between types I and II was analyzed via one-way analysis of variance, *, *P* < 0.05, N.S., not significant.

Then, we spectrophotometrically evaluated catalase activity in bacterial lysates (19). Notably, catalase activity was higher in type I bacterial lysates than in type II lysates in samples cultured in both Sauton and Middlebrook 7H9AD media (Fig. 3a and b). However, there was no significant difference in SOD activity between the two types of bacilli cultured in either medium (Fig. 3c and d), suggesting that BCG Tokyo 172 type I is more tolerant to H_2_O_2_ than type II because of its higher catalase activity. It has been reported that phagocytic cells produce H_2_O_2_ at levels of approximately 0.2 nmol/min/10^6^ cells (20). In this study, the expression of *katG*, *ahpC*, *sodA*, and *tyrA* (encoding thioredoxin) was increased by at least 8-fold (dashed line indicated on Fig. 2) in type I bacilli after exposure to 0.3 mM H_2_O_2_ for 30 min (Fig. 2a, b, c, and e), suggesting that these antioxidative molecule contribute to the tolerance of type I bacilli to H_2_O_2_ stress. Conversely, the induction of these genes was observed after 5 min of exposure to 0.3 mM H_2_O_2_ in type II bacilli (Fig. 2), in line with the viability of type II bacilli after exposure to H_2_O_2_ (Fig. 1).

These data suggest that type I bacilli are more tolerant to H_2_O_2_ stress than type II bacilli *in vitro* because of the stronger induction of intracellular catalase production in type I bacilli.

### The *in vivo* oxidative stress tolerance and induction of Th1 type cytokines in BCG Tokyo 172 types I and II.

**FIG 4:**
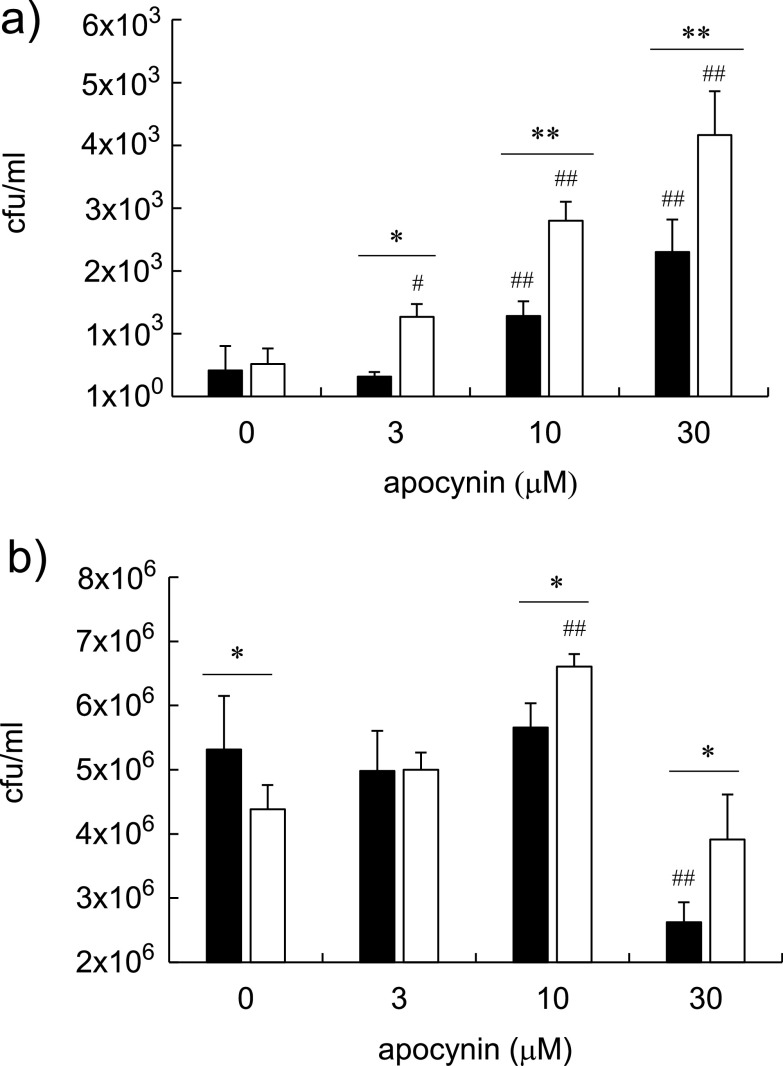
Effect of apocynin on the bactericidal activity of reactive oxygen species in THP-1 and RAW264.7 macrophages. THP-1 cells were differentiated with phorbol 12-myristate 13-acetate. THP-1 human macrophages (a) and RAW264.7 mouse macrophages (b) were infected with Mycobacterium bovis bacillus Calmette-Guérin (BCG) Tokyo 172 type I (solid column) or type II (open column) at a multiplicity of infection of 0.1 to 0.2 and 2 to 5, respectively, and incubated for 24 h in the presence or absence of apocynin. The intercellular bacilli were harvested with hypotonic solution, and then a 10-fold dilution of each solution was plated on Middlebrook 7H11 agar. After 2 or 3 weeks, the colony numbers were counted. The data are representative of three independent experiments, and data are presented as mean and standard deviation. Statistical significances were examined using two-way analysis of variance: type I versus type II, *, *P < *0.05, **, *P < *0.01; versus no apocynin, ^#^, *P < *0.05, ^##^, *P < *0.01.

ROS production is enhanced in host cells following exposure to BCG (21, 22). Thus, we compared the survivability of BCG types I and II in macrophages. THP-1 and RAW264.7 macrophages were incubated with type I or type II BCG in the presence or absence of apocynin, an inhibitor of NADPH oxidase. As the concentration of apocynin was increased, the viability of type II BCG increased more strongly than that of type I BCG in THP-1 and RAW264.7 macrophages (Fig. 4a and b). Apocynin was toxic to RAW264.7 macrophage at 30 μM; however, a significant difference in colony number was observed between bacilli types I and II (Fig. 4b). These data suggest that the activity of NADPH oxidase and its generated ROS in macrophages play important roles in killing BCG bacilli in host cells. In addition, type II bacilli are more susceptible to oxidative stress *in vitro* and *in vivo* because of their lower catalase activity, which might explain their survival in macrophages.

**FIG 5:**
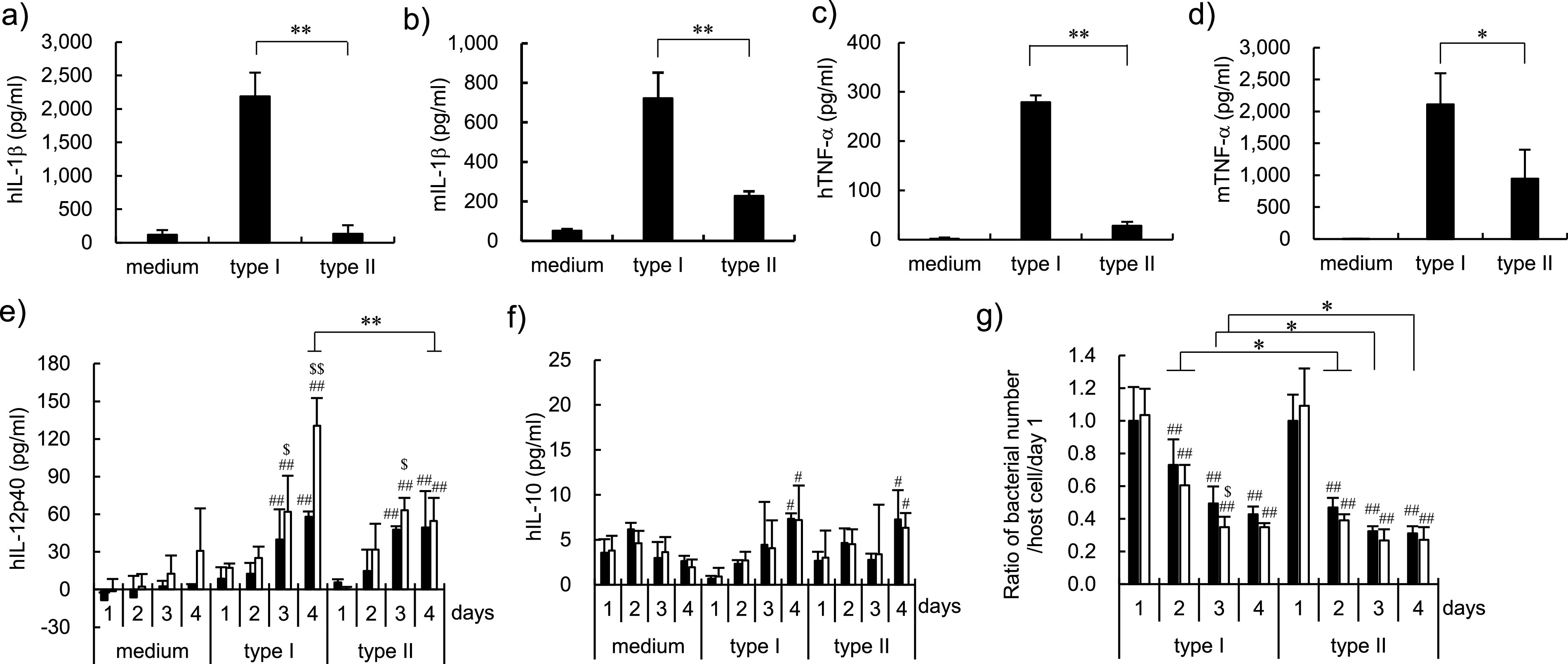
Induction of cytokine secretion by THP-1 and RAW264.7 macrophages after infection with Mycobacterium bovis bacillus Calmette-Guérin (BCG) Tokyo 172 types I and II. THP-1 and RAW264.7 macrophages were infected with BCG Tokyo 172 type I or type II for 3 days at a multiplicity of infection of 0.1 to 0.2 and 2 to 5, respectively. After 2 days of incubation, the concentrations of human (a) and mouse (b) interleukin 1β (IL-1β) and human (c) and mouse (d) tumor necrosis factor α (TNF-α) were measured. The time course experiments of IL-12 p40 (e) and L-10 (f) production by THP-1 cells infected with type I or type II bacilli in the absence (solid column) or presence (open column) of human interferon γ (IFN-γ) (200 U/ml). The cytokine levels in culture supernatants were measured using enzyme-linked immunosorbent assay. (g) The ratio of the number of bacteria per host cell of day 1. The intercellular bacilli were harvested with hypotonic solution, and then a 10-fold dilution of each solution was plated on Middlebrook 7H11 agar. After 2 or 3 weeks, the colony numbers were counted. The data are representative of three independent experiments and the results are presented as mean and standard deviation. Statistical significance was analyzed via three-way analysis of variance: type I versus type II, *, *P* < 0.05, **, *P* < 0.001; versus day 1, ^#^, *P < *0.05, ^##^, *P < *0.01; absence versus presence of IFN-γ, ^$^, *P* < 0.05, ^$$^, *P* < 0.01.

Further, we investigated the abilities of BCG Tokyo 172 types I and II to induce the secretion of Th1 type cytokines, which play pivotal roles in protection against M. tuberculosis infection (23), by THP-1 and RAW264.7 macrophages after infection of these bacilli. After 2 days of incubation with bacilli, interleukin 1β (IL-1β) and tumor necrosis factor α (TNF-α) concentrations were determined by enzyme-linked immunosorbent assay (ELISA). The production of IL-12 p40 by type I bacillus-infected THP-1 macrophages was higher than that by cells infected with type II bacilli in a time-dependent manner (Fig. 5e), whereas no significant difference in IL-10 was observed between macrophages infected with bacilli types I and II (Fig. 5f). A significantly higher number of intracellular type I bacteria was observed in infected macrophages than that of intracellular type II bacilli after 2 days of infection (Fig. 5g). These data indicate that type I bacilli could more strongly induce Th1 type immune responses to macrophages than type II bacilli. The survival ability in the host cell may be related to the difference of induction activity of these cytokines by the macrophages.

### The ratio of BCG types I and II in all commercial lots of Tokyo 172 over a 3-year period.

Honda et al. reported that BCG Tokyo 172 vaccine forms two types of colonies with different morphologies (S and R) and investigated the differences of their genotypes (15). A 22-bp deletion in Rv3405c of the RD16 region was observed in type I S colonies but not in type II R colonies (15). Thus, we investigated the ratio of bacilli types I and II in all lots produced by Japan BCG Laboratory between 2014 and 2016 (Table S1). The average proportions of types I and II were 97.6% ± 1.5% and 2.4% ± 1.5%, respectively (Table 1). Shibayama et al. investigated the content of bacilli types I and II in vaccine seed and commercial lots and detected the aforementioned 22-bp deletion in the RD16 region (24). The relative proportions of type II bacilli in the master and two secondary seed lots were 55.1, 19.5, and 3.6%, respectively, whereas the type II contents of four commercial lots were 1.5, 4.5, 7.4, and 4.3%, respectively. Our study confirmed the previous finding.

**TABLE 1:** The ratio of type I and type II in all commercial lots of BCG Tokyo 172 for 3 years^*a*^

Manufacture yr	No. of lot	Application	Type I (%)	Type II (%)
2014	27	TB vaccine	98.2 (±1.2)	1.8 (±1.2)
	14	Cancer therapy	97.4 (±1.7)	2.6 (±1.8)
2015	21	TB vaccine	98.2 (±1.3)	1.8 (±1.3)
	11	Cancer therapy	97.4 (±1.8)	2.6 (±1.8)
2016	18	TB vaccine	96.8 (±1.2)	3.2 (±1.2)
	9	Cancer therapy	97.9 (±1.3)	2.1 (±1.3)
Total	100		97.6 (±1.5)	2.4 (±1.5)

a The results of the ratio of all lots produced by Japan BCG laboratory from 2014 to 2016 are listed in Table S1.

10.1128/mSphere.00111-21.1 TABLE S1 The proportion of Mycobacterium bovis bacillus Calmette-Guérin (BCG) Tokyo 172 types I and II in all vaccine lots over 3 years. Download Table S1, DOCX file, 0.02 MB. Copyright © 2021 Taniguchi et al. 2021 Taniguchi et al. https://creativecommons.org/licenses/by/4.0/ This content is distributed under the terms of the Creative Commons Attribution 4.0 International license.

## DISCUSSION

Honda et al. observed that during culture, type I bacilli grow faster than type II bacilli, in line with our findings (data not shown). The differences in terms of oxidative stress tolerance were expected to correlate with the growth rate of each type of BCG Tokyo 172. Indeed, type II BCG was more susceptible to H_2_O_2_ than type I BCG. Furthermore, type I bacilli had higher catalase/peroxidase activity than type II bacilli, in line with the induction of *katG* and *ahpC* by H_2_O_2_. OxyR is a sensor protein containing Cys which is oxidized by H_2_O_2_ and upregulates the expression of antioxidative stress genes, including catalase/peroxidase, in Escherichia coli and Salmonella enterica serovar Typhimurium (25–27). In our study, the orthologue of *oxyR* was upregulated by H_2_O_2_ exposure (data not shown); the orthologue was inactivated by multiple mutation in Mycobacterium tuberculosis complex (M. bovis, M. africanum, and M. microti) (28, 29). FurA is a homolog of the ferric uptake regulator Fur which is encoded by a gene located immediately upstream of *katG* in several mycobacterial species, including M. tuberculosis, M. leprae, and M. marinum (30–32). SOD is another antioxidant enzyme, and *sodA* expression has been reported to be enhanced by H_2_O_2_ exposure and nutrient starvation in M. tuberculosis (12). In our study, *sodA* mRNA expression was also enhanced by H_2_O_2_ exposure. Fur or Fur homologs regulate *sodA* in response to oxidative stress in E. coli (33, 34). In our study, *furA* was not upregulated by H_2_O_2_ exposure (data not shown); thus, other regulators might contribute to the expression of *katG*, *ahpC*, and *sodA*.

A number of other proteins are involved in the response to oxidative stress in M. tuberculosis. The thioredoxins TrxA and TrxB are detoxifying antioxidant molecules in M. tuberculosis (35, 36), whereas DosS-DosT/DosR act as redox sensors during latent infection and in dormant mycobacteria (37). WhiB3, which was initially reported to be essential for sporulation in *Streptomyces*, contains Fe-S clusters and responds to oxidative and nitrosative stress in M. tuberculosis (38). It has also been reported to be involved in the survival of this bacterium in macrophages (39). Further, the iron-dependent regulator IdeR is known to be involved in the response to ROS and reactive nitrogen species (RNS) (40), whereas the product of the alkyl hydroperoxide reductase gene (*ahpC*) of M. tuberculosis is considered to protect the organism against oxidative and nitrosative stresses encountered within infected macrophages. After 0.3 mM H_2_O_2_ exposure for 30 min, the mRNA levels of antioxidative stress response genes were significantly different between the two bacillus types, suggesting that the difference in the antioxidative activity reflects the differential growth of the two types of BCG bacilli *in vitro*.

ROS and RNS are crucial molecules in the response to mycobacterial and other bacterial infections in host cells (41). M. tuberculosis complex, including M. bovis, consists of intracellular pathogens, and phagocytic cells such as macrophages play a crucial role in eliminating the bacilli. Their killing mechanisms include superoxide generation by phagocyte NADPH oxidase (42). The study results illustrated that type II BCG are more sensitive to H_2_O_2_ and the oxidative molecules generated by host cells *in vitro* than type I bacilli, and this difference in terms of antioxidative activity between BCG types I and II should reflect their viability within host cells.

Jain et al. reported that the overexpression of an antioxidative molecule in BCG augmented its protective effect against tuberculosis by modulating innate and adaptive immune responses (43). We previously investigated Th1 cytokine induction by 13 BCG substrains (2). The early shared strains such as BCG-Russia, BCG-Japan (Tokyo 172), and BCG-Moreau exhibited higher cytokine induction activity in macrophages, including IL-1 and TNF-α (2). In this study, the accumulation of IL-1β, TNF-α, and IL-12 p40 in the supernatant of type I BCG-infected macrophages was higher than that in the supernatant of type II BCG-infected macrophages. IL-1 is known to induce and enhance memory-type immune responses (44–46), whereas TNF-α protects against *Mycobacterium* infection (47) and plays a functional role in CD4 and CD8 T cell responses to M. tuberculosis infection (48). IL-12 prevents the growth of M. tuberculosis bacilli in lungs via the induction, expansion, and control of effector T cell responses through the production of cytokines, such as IL-17, IL-23, and IFN-γ (49, 50). BCG bacilli induced the production of proinflammatory cytokines, including TNF-α and IL-12, by macrophage (51). The levels of these cytokines were measured as mediators responsible for the protective activity of vaccines against infection by M. tuberculosis bacilli (52–54). In our study, type I bacilli induced IL-1β, TNF-α, and IL-12 p40 production by macrophages more strongly than type II bacilli. The results suggest that type I bacilli exhibit better properties for tuberculosis vaccines than type II bacilli.

Taken together, the differences of the *in vitro* and *in vivo* antioxidative stress properties of bacilli types I and II could contribute to the production of proinflammatory cytokines to protect against infection by M. tuberculosis. In particular, the bacilli exhibited differences in catalase/peroxidase activity, which could explain their different protective effects against infection. The proportion of bacilli types I and II in all commercial lots of BCG Tokyo 172 over 3 years (100 lots) was constant at 97.6% ± 1.5% and 2.4% ± 1.5%, respectively. Overall, the current findings provide basic yet indispensable information for evaluating the quality of BCG Tokyo 172 vaccine lots. The findings also highlighted the importance of analyzing the content of vaccine lots for quality control during vaccine production.

## MATERIALS AND METHODS

### Bacterial cultures and frozen stock.

M. bovis BCG-Japan (Tokyo 172), Tokyo 172 type I, and Tokyo 172 type II were provided from Japan BCG Laboratory (Tokyo, Japan).

Bacterial cultures were prepared in Middlebrook 7H9 broth (Difco, Detroit, MI, USA) supplemented with 10% AD (5% bovine serum albumin [fraction V] and 2% dextrose) and 0.05% Tween 80 or in Sauton medium, and they were grown at 37°C under static conditions. Bacteria were grown to an optical density of 0.6 to 0.8 at 530 nm. Further, the cultures were aliquoted and stored at −70°C until needed. The number of CFU in the aliquots was determined by colony assays on Middlebrook 7H11 agar (Difco) supplemented with 10% oleic acid-albumin-dextrose-catalase (OADC; 0.05% oleic acid, 5% bovine serum albumin [fraction V], 2% dextrose, and 0.004% bovine liver catalase; Difco).

### Catalase and SOD activity measurements.

Catalase activity was by a conventional method as described previously (3). Briefly, bacilli types I and II were cultured on Ogawa medium in a test tube for 4 weeks. The detection agents 5% Tween 80 and 15% H_2_O_2_ were added to the test tube, and the foam height was measured from the surface of the medium after 5 min at room temperature. Intracellular catalase activity in the bacillus lysates was measured as the reduction in optical density at 240 nm resulting from the conversion of H_2_O_2_ to water, as previously described (19). The bacterial cells from the bacterial frozen bacterial stock were harvested and beaten with 0.1 mm zirconia/silica beads (BioSpec Products, Inc., Bartlesville, OK, USA). Further, 10 mM H_2_O_2_ was added, and its degradation was measured at 240 nm every 10 s using a spectrophotometer (U-2000, Hitachi High-Tech Science Co., Tokyo, Japan). In the cell lysates, catalase units were calculated in comparison to a known catalase standard. SOD activity in the lysate was measured using a WST SOD assay kit (Dojindo Laboratories, Kumamoto, Japan).

### RNA extraction and quantitative reverse transcriptase PCR.

RNA was extracted from M. bovis BCG using TRIzol Max Bacterial RNA isolation kit (Invitrogen, Life Technologies Japan, Tokyo, Japan). Further, the quantity and quality of the extracted RNA were determined using NanoVue plus spectrophotometer (GE Healthcare, Chicago, IL, USA). For cDNA synthesis, 40 ng of RNA were used with PrimeScript RT master mix (TaKaRa, Shiga, Japan). Real-time PCRs were performed in an Applied Biosystems StepOnePlus real-time PCR system with real-time PCR software version 2.3 (Applied Biosystems, Thermo Fisher, Foster City, CA, USA). Each reaction was performed in a total volume of 10 μl on a 48-well optical reaction plate (Applied Biosystems) containing 5 μl of TB Green Fast qPCR Mix (TaKaRa), 500 to 1,000 ng of cDNA (1/10 dilution), and two gene-specific primers at a final concentration of 0.2 μM each. The real-time cycling conditions were as follows: (i) 95°C for 30 s and (ii) 40 cycles at 95°C for 5 s and 60°C for 10 s. Notably, melting curve analysis verified that each reaction contained a single PCR product. Reported gene expression levels were normalized to transcripts of 16S rRNA. The primers used for real-time PCR are indicated in Table 2.

**TABLE 2:** Primers used for real-time PCR

Antioxidative stress response genes		
*ahpC*	5′-AAGGTCGACGCCAAGCAG-3′	5′-TTGAGCTTGCTGAACGCCG-3′
*dosR*	5′-CAGAGGTGTAGGACGTGAGG-3′	5′-GATGTCGCGGTGCTGGAT-3′
*dosS*	5′-AGCCGAGTGATACCCTGCGA-3′	5′-CTGGCTTTGCAGGGTGCT-3′
*dosT*	5′-AGGTCGAAGATCGCAGATCG-3′	5′-TGCATGACCACGTCATCCA-3′
*katG*	5′-ATGCTGGCCACTGACCTCTC-3′	5′-ATGTCTCGGTGGATCAGCTTG-3′
*ideR*	5′-AGCACGTGATGAGCGAGGA-3′	5′-TCGGTCAACCGGACCAGGTT-3′
*sodA*	5′-TTTCAACCTCGCCGGCCA-3′	5′-GGAACTGCGCACGGAACT-3′
*sodC*	5′-GCAGGTACGCGGTGACGGTT-3′	5′-CGTAGCGTTCTGGCGGAATG-3′
*trxA*	5′-ACTTTTGGGCACCGCTGTG-3′	5′-CGAGGCCAGATCTTTCTCGGT-3′
*trxB*	5′-AACAGCAGTGCCTACGCTGG-3′	5′-CGGAGCCGATAACGATCACGTC-3′
*whiB3*	5′-GCGGCATGGACTCATCGATG-3′	5′-CATAGGGCTCACCGACCTCT-3′
16S rRNA	5′-CAACGCGAAGAACCTTACCT-3′	5′-TGCACACAGGCCACAAGGGA-3′

BCG Tokyo 172 subpopulation		
Type I specific	5′-CTCGATCACCTGGGCACTC-3′
Type II specific	5′-CGAAGCTGACCAGACTGTTGC-3′
Common reverse	5′-GGTCGAGAGCCCGTTCG-3′

### Survival of bacilli in macrophages.

The human monocyte cell line THP-1 (ATCC TIB-202) and mouse macrophage cell line RAW264.7 (ATCC TIB-71) were purchased from American Type Culture Collection (Manassas, VA, USA). Cells were maintained in RPMI 1640 or Dulbecco’s Modified Eagle Medium (Wako Pure Chemical Industries, Ltd., Osaka, Japan) supplemented with 5% heat-inactivated fetal bovine serum (FBS), 100 U/ml penicillin G (Meiji Seika Pharma Co., Ltd., Tokyo, Japan), and 100 μg/ml streptomycin (Meiji Seika Pharma) in a humidified 5% CO_2_ atmosphere at 37°C. The medium was replaced with medium containing 100 U/ml penicillin G, an antibiotic with no significant antimycobacterial activity at this concentration, before cells were infected with BCG bacilli.

The differentiation of THP-1 cells into macrophages was induced by incubation with 10 nM phorbol 12-myristate 13-acetate (PMA) (55). THP-1 cells (2.5 × 10^5^ cells/well) were cultured in 1 ml of RPMI 1640 without FBS in a 24-well plate containing 10 nM PMA for 24 h and washed twice with PBS (−), and then the culture medium was replaced with RPMI 1640 containing 5% FBS. THP-1 macrophages or RAW264.7 cells (2 × 10^5^ cells/well in a 24-well plate) were incubated with BCG bacilli (2.5 × 10^6^ CFU/well) for 4 h, and then the cells were washed with PBS (−) and cultured with medium containing 100 μg/ml amikacin for 4 h to kill the outer cellular bacilli. The medium was removed and replaced with RPMI 1640 containing 5% FBS with or without apocynin (4′-hydroxy-3′-methoxyacetophenone, Sigma-Aldrich Co., St. Louis, MO, USA). After 24 h of infection, THP-1 or RAW264.7 macrophages were harvested using stylized water, and the bacilli inside the host cells were collected. The number of live bacteria was counted on Middlebrook 7H11 agar using the colony assay. The multiplicity of infection was calculated as the ratio of the number of bacterial particles to the number of host cells.

### Measurement of cytokine levels in the culture supernatant.

The amounts of human IL-1β, IL-10, IL-12 p40, and TNF-α and mouse IL-1β and TNF-α in the supernatants of THP-1 and RAW264.7 macrophages were quantitated by ELISA using a BD OptEIA ELISA set (BD Bioscience, San Jose, CA, USA).

### Quantification of the composition of type I and type II subpopulations in commercial BCG vaccine lots.

The proportion of bacilli types I and II in each BCG vaccine lot was determined as the ratio of the RD16-deleted genotype, a unique genetic feature of the type I subpopulation, to the intact genotype, corresponding to the type II subpopulation, using specific real-time PCR primers (Table 2), which were described in a previous report (56). The annealing temperatures for types I and II were 60 and 62°C, respectively. The DNA concentration of both types was analyzed using the StepOnePlus real-time PCR system and real-time PCR software version 2.3, in triplicate in three independent experiments.

### Statistical analysis.

Statistical significance among data sets was assessed by analysis of variance using SigmaPlot (Systat Software, Inc., San Jose, CA, USA), and differences were considered significant at a *P *value of <0.05.
