# Supplementary material for: Comparative Study of the Susceptibility to Oxidative Stress between Two Types of Mycobacterium bovis BCG Tokyo 172
Source: mSphere. 2021 Mar 10;6(2):e00111-21. doi: 10.1128/mSphere.00111-21 (PMC8546687; doi:10.1128/mSphere.00111-21)
Supplement: TABLE S1 [file msphere.00111-21-st001.docx]

| **Supplemental Table 1. The proportion of *Mycobacterium bovis* bacillus Calmette–Guérin (BCG) Tokyo 172 types I and II in all vaccine lot of over 3 years** | | | | | | |
| --- | --- | --- | --- | --- | --- | --- |
| Serial No. | | Temporary lot No. | Manufacture year | Application^*1^ | Type I (%) | Type II(%) |
| 1 | 14-1 | | 2014 | T | 97.4 | 2.6 |
| 2 | 14-2 | | 2014 | T | 99.6 | 0.4 |
| 3 | 14-3 | | 2014 | T | 96.6 | 3.4 |
| 4 | 14-4 | | 2014 | T | 98.5 | 1.5 |
| 5 | 14-5 | | 2014 | T | 97.8 | 2.2 |
| 6 | 14-6 | | 2014 | T | 94.4 | 5.6 |
| 7 | 14-7 | | 2014 | T | 98.4 | 1.6 |
| 8 | 14-8 | | 2014 | T | 97.9 | 2.1 |
| 9 | 14-9 | | 2014 | T | 98.4 | 1.6 |
| 10 | 14-10 | | 2014 | T | 98.2 | 1.8 |
| 11 | 14-11 | | 2014 | T | 96.3 | 3.7 |
| 12 | 14-12 | | 2014 | T | 99.2 | 0.8 |
| 13 | 14-13 | | 2014 | T | 99.5 | 0.5 |
| 14 | 14-14 | | 2014 | T | 98.7 | 1.3 |
| 15 | 14-15 | | 2014 | T | 99.2 | 0.8 |
| 16 | 14-16 | | 2014 | T | 98.8 | 1.2 |
| 17 | 14-17 | | 2014 | T | 99.1 | 0.9 |
| 18 | 14-18 | | 2014 | T | 97.2 | 2.8 |
| 19 | 14-19 | | 2014 | T | 97.6 | 2.4 |
| 20 | 14-20 | | 2014 | T | 98.1 | 1.9 |
| 21 | 14-21 | | 2014 | T | 97.9 | 2.1 |
| 22 | 14-22 | | 2014 | T | 99.3 | 0.7 |
| 23 | 14-23 | | 2014 | T | 98.9 | 1.1 |
| 24 | 14-24 | | 2014 | T | 99.0 | 1.0 |
| 25 | 14-25 | | 2014 | T | 96.6 | 3.4 |
| 26 | 14-26 | | 2014 | T | 98.6 | 1.4 |
| 27 | 14-27 | | 2014 | T | 99.4 | 0.6 |
| 28 | 14-28 | | 2014 | B | 95.2 | 4.8 |
| 29 | 14-29 | | 2014 | B | 97.1 | 2.9 |
| 30 | 14-30 | | 2014 | B | 97.7 | 2.3 |
| 31 | 14-31 | | 2014 | B | 97.9 | 2.1 |
| 32 | 14-32 | | 2014 | B | 97.6 | 2.4 |
| 33 | 14-33 | | 2014 | B | 97.6 | 2.4 |
| 34 | 14-34 | | 2014 | B | 99.6 | 0.4 |
| 35 | 14-35 | | 2014 | B | 98.5 | 1.5 |
| 36 | 14-36 | | 2014 | B | 95.1 | 4.9 |
| 37 | 14-37 | | 2014 | B | 99.2 | 0.8 |
| 38 | 14-38 | | 2014 | B | 93.7 | 6.3 |
| 39 | 14-39 | | 2014 | B | 96.7 | 3.3 |
| 40 | 14-40 | | 2014 | B | 99.0 | 1.0 |
| 41 | 14-41 | | 2014 | B | 99.2 | 0.8 |
|  |  | |  | AV | 97.9 | 2.1 |
|  |  | |  | SD | 1.4 | 1.4 |
|  |  | |  |  |  |  |
| 42 | 15-1 | | 2015 | T | 98.4 | 1.6 |
| 43 | 15-2 | | 2015 | T | 99.9 | 0.1 |
| 44 | 15-3 | | 2015 | T | 99.4 | 0.6 |
| 45 | 15-4 | | 2015 | T | 95.1 | 4.9 |
| 46 | 15-5 | | 2015 | T | 96.6 | 3.4 |
| 47 | 15-6 | | 2015 | T | 98.3 | 1.7 |
| 48 | 15-7 | | 2015 | T | 98.8 | 1.2 |
| 49 | 15-8 | | 2015 | T | 96.6 | 3.4 |
| 50 | 15-9 | | 2015 | T | 99.7 | 0.3 |
| 51 | 15-10 | | 2015 | T | 99.2 | 0.8 |
| 52 | 15-11 | | 2015 | T | 97.6 | 2.4 |
| 53 | 15-12 | | 2015 | T | 97.6 | 2.4 |
| 54 | 15-13 | | 2015 | T | 99.0 | 1.0 |
| 55 | 15-14 | | 2015 | T | 97.9 | 2.1 |
| 56 | 15-15 | | 2015 | T | 95.6 | 4.4 |
| 57 | 15-16 | | 2015 | T | 98.4 | 1.6 |
| 58 | 15-17 | | 2015 | T | 99.0 | 1.0 |
| 59 | 15-18 | | 2015 | T | 97.4 | 2.6 |
| 60 | 15-19 | | 2015 | T | 99.4 | 0.6 |
| 61 | 15-20 | | 2015 | T | 98.9 | 1.1 |
| 62 | 15-21 | | 2015 | T | 99.0 | 1.0 |
| 63 | 15-22 | | 2015 | B | 99.0 | 1.0 |
| 64 | 15-23 | | 2015 | B | 97.3 | 2.7 |
| 65 | 15-24 | | 2015 | B | 95.2 | 4.8 |
| 66 | 15-25 | | 2015 | B | 97.8 | 2.2 |
| 67 | 15-26 | | 2015 | B | 93.8 | 6.2 |
| 68 | 15-27 | | 2015 | B | 98.8 | 1.2 |
| 69 | 15-28 | | 2015 | B | 97.0 | 3.0 |
| 70 | 15-29 | | 2015 | B | 95.3 | 4.7 |
| 71 | 15-30 | | 2015 | B | 98.3 | 1.7 |
| 72 | 15-31 | | 2015 | B | 99.2 | 0.8 |
| 73 | 15-32 | | 2015 | B | 99.1 | 0.9 |
|  |  | |  | AV | 97.9 | 2.1 |
|  |  | |  | SD | 1.5 | 1.5 |
|  |  | |  |  |  |  |
| 74 | 16-1 | | 2016 | T | 99.2 | 0.8 |
| 75 | 16-2 | | 2016 | T | 98.3 | 1.7 |
| 76 | 16-3 | | 2016 | T | 96.6 | 3.4 |
| 77 | 16-4 | | 2016 | T | 96.3 | 3.7 |
| 78 | 16-5 | | 2016 | T | 97.0 | 3.0 |
| 79 | 16-6 | | 2016 | T | 96.6 | 3.4 |
| 80 | 16-7 | | 2016 | T | 97.4 | 2.6 |
| 81 | 16-8 | | 2016 | T | 97.4 | 2.6 |
| 82 | 16-9 | | 2016 | T | 96.5 | 3.5 |
| 83 | 16-10 | | 2016 | T | 98.0 | 2.0 |
| 84 | 16-11 | | 2016 | T | 95.7 | 4.3 |
| 85 | 16-12 | | 2016 | T | 95.6 | 4.4 |
| 86 | 16-13 | | 2016 | T | 96.0 | 4.0 |
| 87 | 16-14 | | 2016 | T | 95.7 | 4.3 |
| 88 | 16-15 | | 2016 | T | 95.4 | 4.6 |
| 89 | 16-16 | | 2016 | T | 99.2 | 0.8 |
| 90 | 16-17 | | 2016 | T | 95.3 | 4.7 |
| 91 | 16-18 | | 2016 | T | 96.4 | 3.6 |
| 92 | 16-19 | | 2016 | B | 99.6 | 0.4 |
| 93 | 16-20 | | 2016 | B | 98.0 | 2.0 |
| 94 | 16-21 | | 2016 | B | 96.8 | 3.2 |
| 95 | 16-22 | | 2016 | B | 97.1 | 2.9 |
| 96 | 16-23 | | 2016 | B | 98.7 | 1.3 |
| 97 | 16-24 | | 2016 | B | 98.9 | 1.1 |
| 98 | 16-25 | | 2016 | B | 99.2 | 0.8 |
| 99 | 16-26 | | 2016 | B | 95.9 | 4.1 |
| 100 | 16-27 | | 2016 | B | 97.1 | 2.9 |
|  |  | |  | AV | 97.2 | 2.8 |
|  |  | |  | SD | 1.3 | 1.3 |
| *1, T; trans dermal, B; bladder infusion | | | |  |  |  |

|  |  |  |  |  |  |
| --- | --- | --- | --- | --- | --- |
